# Complications Related to Esophageal Stent (Boston Scientific Wallflex vs. Merit Medical Endotek) Use in Benign and Malignant Conditions

**DOI:** 10.7759/cureus.7380

**Published:** 2020-03-23

**Authors:** Rajesh Essrani, Hiral Shah, Shashin Shah, Jennifer Macfarlan

**Affiliations:** 1 Internal Medicine, Geisinger Medical Center, Danville, USA; 2 Internal Medicine, Lehigh Valley Health Network, Allentown, USA; 3 Gastroenterology, Lehigh Valley Health Network, Allentown, USA; 4 Public Health, Lehigh Valley Health Network, Allentown, USA

**Keywords:** esophageal stent, boston scientific, merit medical endotek, wallflex, benign esophageal condition, malignant esophageal condition, chest pain, stent migration, esophageal cancer, esophageal leak

## Abstract

Background

In our institutions, there are two types of stents used: the Boston Scientific Wallflex (Marlborough, Massachusetts) and Merit Medical Endotek (South Jordan, Utah). So we performed this retrospective study to compare complication rates in various esophageal disorders to improve our quality of care.

Methods

Charts were reviewed to capture gender, indications of stent placement, stent length/diameter, age of the patient at the time of stent placement, length of hospital stay, physicians performing a procedure, and complications within 90 days of stent placement.

Results

A total of 67 patients (71.6% male) underwent stent placement (WallFlex 49.3% and Merit 50.8%) for malignant (68.7%) mainly esophageal obstruction by primary esophageal cancer (89.1%) and benign causes (31.3%) mainly esophageal leak (66.7%). Merit and WallFlex used in malignant conditions were 82.4% and 54.6%, respectively, and in benign conditions, they were 17.7% and 45.5%, respectively. The mean age at which endoscopy was performed was 64. Complications post Merit and WallFlex placement were 79.4% and 60.6%, respectively. Complications with malignant and benign conditions were 73.9% and 61.9%, respectively. Complications with 19, 18, and 23 mm diameters were 75.0%, 66.7%, and 69.4%, respectively. Complications with 120, 150, 100, 15, 12, 10 mm stent lengths were 84.6%, 58.3%, 58.8%, 80.0%, 75.0%, and 33.3%, respectively.

Conclusion

Our study showed that the Merit stent was mainly used, and the major indication of stent placement was a malignant condition. Major complications were seen when the reason for stent placement was a malignant condition, the diameter was 19 mm, the length was 120 mm, and the use of the Merit stent.

## Introduction

An esophageal stent is a minimally invasive procedure that is often used in benign esophageal disorders, such as esophageal leaks, fistulas, refractory strictures, and malignant conditions, such as locally unresectable or advanced metastatic cancer of the esophagus, those with poor functional status who can’t tolerate surgery or chemoradiotherapy, or those in whom previous treatment failed or those with locally recurrent disease [1].

There are numerous treatment options for malignant and benign esophageal conditions like surgical resection, photodynamic therapy, thermal ablative therapy, chemotherapy-radiation therapy, and esophageal stent. Endoscopic stenting offers significant advantages over these other therapies. Stents are generally easy and quick to place and safe, with a good complication profile.

There are a variety of a self-expandable plastic and self-expandable metal stents available in the United States. Esophageal stents that are mostly used in the U.S. include Boston Scientific (Marlborough, Massachusetts), Cook Medical (Bloomington, Indiana), EndoChoice (Alpharetta, Georgia), Merit Medical Endotek (South Jordan, Utah), and Taewoong Medical Co (Seoul, Korea). These stents are also available in three types: uncovered, fully covered (FC), and partially covered (PC).

There are various complications associated with stent placement, which can be divided into:

Immediate (at the time of placement) such as aspiration, airway compromise, malposition, delivery stent entrapment, stent dislodgement, and perforation.

Early (up to one week after stent placement) such as bleeding, chest pain, and nausea.

Late (beyond one week of successful stent placement) such as recurrent dysphagia due to re-obstruction from a tumor or food impaction, migration, bleeding, tracheoesophageal fistula, and gastroesophageal reflux disease/aspiration.

These complications depend on multiple factors like an indication of stent placement, location of tumor, use of chemoradiation before stent placement, presence or absence of a fistula or tumor shelf, tumor vascularity, and the diameter/design of the prosthesis [2-19]. Esophageal stenting aims to restore luminal patency and thereby nutritional intake and improve quality of life [20].

In our institutions, there are two types of stents used: Boston Scientific Wallflex and Merit Medical Endotek. So we performed this retrospective study to compare complication rates in various esophageal disorders to improve our quality of care.

This article was presented as a poster (Essrani R, Shah H, Shah S, Macfarlan J: Su1149 Complications Related to Esophageal Stent (Boston Scientific [Wallflex] Vs Merit Medical Endotek) Use in Benign and Malignant Condition - A Single Center Retrospective Review. Gastrointest Endosc. 2018, 87: AB294). The abstract is taken from the published article.

## Materials and methods

Background

Multiple studies have compared esophageal stents and complications.

Siersema et al. performed a randomized trial on 100 patients with dysphagia caused by esophagogastric carcinoma using the PC Ultraflex stent (Boston Scientific), the PC Flamingo Wallstent (Boston Scientific), and the FC Gianturco Z stent (Wilson-Cook, Denmark). The three stents were equally effective in improving the dysphagia score without a significant difference in the major complication rate (Ultraflex stent 24%, Flamingo Wallstent 18%, and Gianturco Z stent 36%) [13].

Conio et al. randomized 101 patients to a Polyflex (Boston Scientific) or Ultraflex stent (Boston Scientific), showing similar effectiveness in the palliation of dysphagia but higher rates of complication post the Polyflex stent [14].

Verschuur et al. randomized 125 patients to an Ultraflex stent (Boston Scientific), FC double-layered Niti-S stent (Taewong Medical), and the Polyflex stent (Boston Scientific). The Ultraflex and Niti-S stents were equally effective with equal overall complication rates, but recurrent dysphagia occurred more frequently with the Ultraflex stent (52 % vs. 31%). The Polyflex SEPS was associated with high failure of stent placement (17%) and increased migration risk [21].

Dua et al. performed a prospective study using the FC Polyflex stent in refractory benign strictures. Polyflex stents were associated with frequent complications such as severe chest pain, bleeding, perforation, bleeding, reflux, food impaction, and fistula formation [22]. These serious side effects were confirmed in other retrospective series with high migration and complication rates (respectively 7-66 and 6-28%) [23-27].

May et al. compared the uncovered Ultraflex, partially covered and uncovered Wallstent, and partially covered Z stent in 96 patients with inoperable esophageal cancers. The improvement in the degree of dysphagia and complication rates was similar across the different stent groups [28].

Aims/objectives of the study

The purpose of this study was to assess the complication rates in both benign and malignant conditions between the Boston Scientific Wallflex and the Merit Medical Endotek stents. The study aimed to show that complication rates for bleeding, stent migration, and chest pain vary for different indications of stent placement, different size stents, and different types of stents.

Methods

This retrospective chart review collected data on 67 patients who underwent esophageal stent placement for benign and malignant esophageal conditions between January 1, 2006, and December 12, 2016, at Lehigh Valley Health Network (Cedar Crest campus), Pennsylvania, USA. The study was approved by the Institutional Review Board (LVHN IRB Number: PRO00004894). The requirement of informed consent was waived at the time of approval due to the retrospective design.

Patients were included in the study if they were above 18 years of age and underwent esophageal stent placement for a malignant or benign cause. Five cohorts were created based on stent placement. Cohort A-: esophageal obstruction due to primary cancer, Cohort B: esophageal obstruction due to the secondary tumor, Cohort C: esophageal stricture, Cohort D: esophageal leak or perforation, and Cohort E: tracheoesophageal fistula. The two types of stents used were the Boston Scientific WallFlex and Merit Medical Endotek.

The stents used had various diameters and lengths, all of which were noted. There were three common complications following stent placement: chest pain, bleeding, and stent migration. Some of the complications developed immediately post stent placement but others took time. Patient charts were followed for 90 days after their first stent placement to capture complications. Chest pain was usually not quantified in charts, so no scale (ex. 1-10) was used. Patient age at the time of stent placement was calculated using the date of birth and date of stent placement. The length of hospital stay was also calculated using the date of endoscopic stent placement and hospital discharge date. Other collected variables included gender and the person performing stent placement.

Statistical analysis

Categorical variables were described using frequencies and percentages. Continuous variables were described using the median and interquartile range. The overall complication was determined by whether or not the patient had at least one of the captured complications. All analyses were done using SAS 9.3 (SAS Institute, Cary, NC).

## Results

A total of 67 patients underwent stent placement, of which 71.6% were male and 28.4% were female. The median age at which endoscopy was performed was 64 years. Just under half (49.3%) used the WallFlex stent and just over half (50.8%) used the Merit stent (Table 1). Malignant causes made up 68.7% of the reasons for stent placement, largely due to esophageal obstruction by primary esophageal cancer (89.1%) and benign causes made up 31.3% of the reasons for stent placement, largely due to an esophageal leak (66.7%) (Table 2). One provider performed 62.7% of procedures, while the other performed 37.3% of endoscopic procedures. In malignant conditions, Merit and WallFlex were used 60.9% and 39.1% of the time, respectively, and in benign conditions, were used 28.6% and 71.4%, respectively. With regard to the stent itself, the most common diameter used was 23 mm (74.2%) followed by 18 mm (13.6%) and 19 mm (12.1%). The most common length used was 120 mm (38.8%) followed by 100 mm (25.4%), 150 mm (17.9%), 15 mm (7.5%), 12 mm (6.0%), and 10 mm (4.5%).

**Table 1:** Study outcomes for the sample as a whole and stratifies by indication. Data are n (%) unless otherwise stated. Data might not add to 100% due to rounding. IQR= Interquartile range.

Outcomes	Total (n=67)	Malignant (n=46)	Benign (n=21)
Gender			
Male	48 (71.6)	35 (76.1)	13 (61.9)
Female	19 (28.4)	11 (23.9)	8 (38.1)
Age at time of endoscopy, years median (IQR)	64.0 (58.0-69.0)	66.0 (60.0-69.0)	59.0 (48.0-68.0)
Endoscopist			
Endoscopist 1	25 (37.3)	18 (39.1)	7 (33.3)
Endoscopist 2	42 (62.7)	28 (60.9)	14 (66.7)
Manufacturer			
Boston Scientific (Wallflex)	33 (49.3)	18 (39.1)	15 (71.4)
Merit Medical Endotek	34 (50.8)	28 (60.9)	6 (28.6)
Diameter (n=66)			
18	9 (13.6)	6 (13.3)	3 (14.3)
19	8 (12.1)	7 (15.6)	1 (4.8)
23	49 (74.2)	32 (71.1)	17 (81.0)
Length			
10	3 (4.5)	2 (4.4)	1 (4.8)
12	4 (6.0)	2 (4.4)	2 (9.5)
15	5 (7.5)	4 (8.7)	1 (4.8)
100	17 (25.4)	12 (26.1)	5 (23.8)
120	26 (38.8)	21 (45.7)	5 (23.8)
150	12 (17.9)	5 (10.9)	7 (33.3)
Length of stay, days median (IQR)	2.0 (1.0-6.0)	2.0 (1.0-4.0)	8.0 (1.0-17.0)
Complication			
Yes	47 (70.2)	34 (73.9)	13 (61.9)
No	20 (29.9)	12 (26.1)	8 (38.1)
Chest pain			
Yes	39 (58.2)	28 (60.9)	11 (52.4)
No	28 (41.8)	18 (39.1)	10 (47.6)
Bleeding			
Yes	9 (13.4)	8 (17.4)	1 (4.8)
No	58 (86.6)	38 (82.6)	20 (95.2)
Migration			
Yes	16 (23.9)	11 (23.9)	5 (23.8)
No	51 (76.1)	35 (76.1)	16 (76.2)
Number of complications median (IQR)	1.0 (0.0-2.0)	1.0 (0.0-2.0)	1.0 (0.0-1.0)

**Table 2:** Reasons for stent placement

Indication	Frequency (Percent)
Esophageal obstruction due to primary cancer	41 (61.2)
Esophageal obstruction due to secondary tumor	1 (1.5)
Esophageal stricture	5 (7.5)
Esophageal leak/perforation	14 (20.9)
Tracheoesophageal fistula	2 (3.0)
Esophageal obstruction due to primary cancer and esophageal stricture	4 (6.0)

Within 90 days post-stent placement, 70.2% of patients experienced at least one complication, with chest pain (58.2%) being the most common complication followed by stent migration (23.9%) and bleeding (13.4%).

When grouped by the Merit and WallFlex stent types, complications occurred in 79.4% and 60.6% of patients, respectively, with chest pain (67.7% and 48.5%) being the most common complaint followed by stent migration (29.4% and 18.2%) and bleeding (14.7% and 12.1%). Complications within malignant and benign conditions were 73.9% and 61.9%, respectively.

Complications with 18, 19, and 23 mm diameters were 66.7%, 75.0%, and 69.4%, respectively. Complications with 150, 120, 100, 15, 12, and 10 mm stent lengths were 58.3%, 84.6%, 58.8%, 80.0%, 75.0% and 33.3%, respectively.

**Figure 1:**
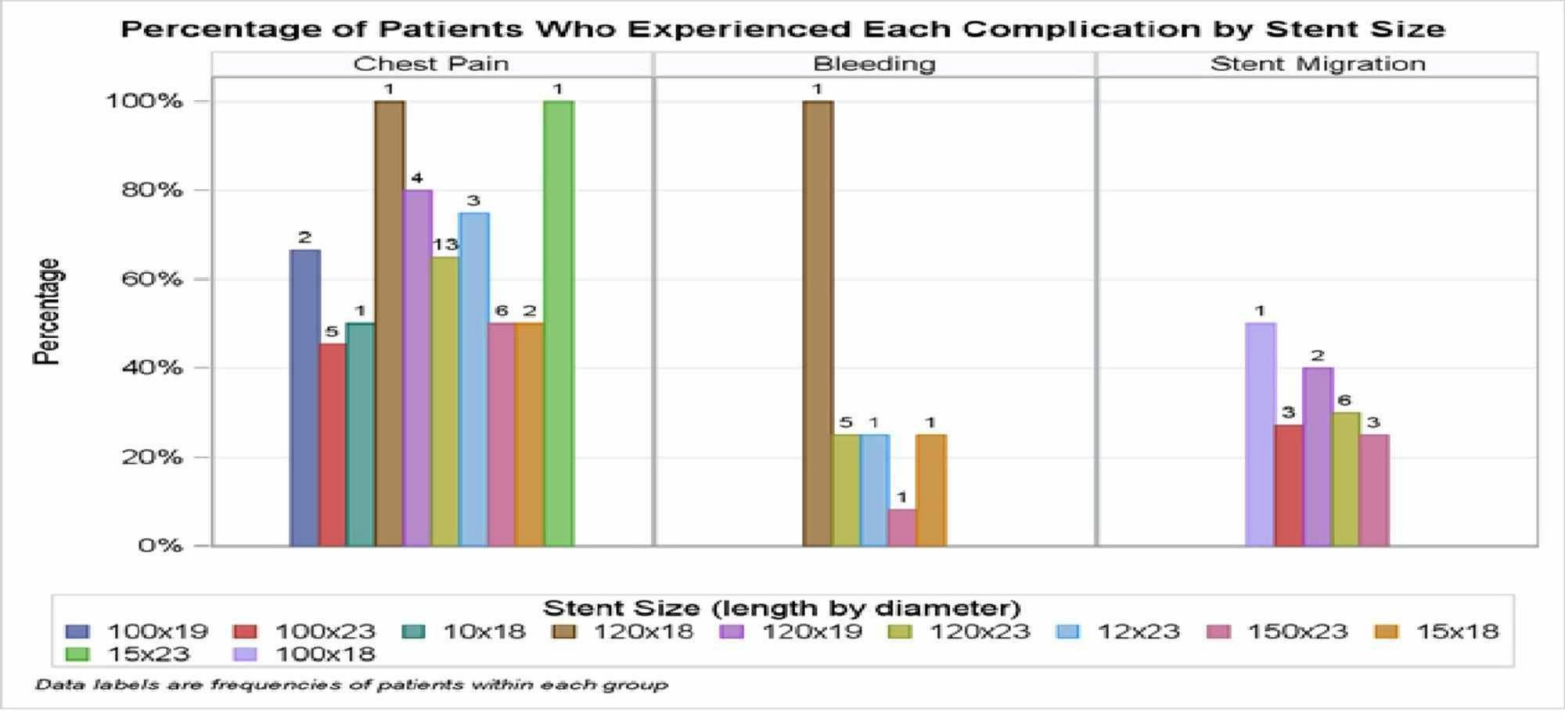
Percentage of patients who experienced each complication by stent size

The most common stent used in our institute was 120 X 23 in size, so it has the most common complication: chest pain followed by stent migration and bleeding (Figure 1).

## Discussion

The main indication for stent placement in our experience was for the palliation of dysphagia secondary to obstructing esophageal cancer followed by an esophageal leak.

Numerous treatment options are available to help in the palliation of a malignant esophageal obstruction such as photodynamic therapy, thermal ablative techniques, surgical resection or bypass, and chemotherapy/radiotherapy [29-30]. Endoscopic stenting offers significant advantages over these other therapies. Stents are generally easy and quick to place, safe, with a good complication profile, and well-tolerated with a low incidence of significant pain.

The most common complication we encountered was chest pain, which was transient and mostly resolved without any intervention. Notably, we did not encounter any perforations related to stent placement. For comparison, other recent series report perforation rates of 0% to 6% and procedure-related mortality rates of 2% to 4% [14]. There was an increased risk of significant complications (chest pain, bleeding, and stent migration) with the large-diameter stents as compared with smaller-diameter prostheses. This is seen in other studies [19]. The major complication rate was seen in malignant conditions as compared to benign. Merit is mostly used in malignant conditions as compared to WallFlex (82.4% Vs. 54.6%), therefore, more complications were observed post Merit placement with chest pain being the most common complaint followed by stent migration and bleeding.

The major drawback of this study includes retrospective nature, so increasing the risk of selection bias and missing minor complications post stent placement. The sample size was small.

There have been no head-to-head studies done to compare the Merit and WallFlex stents, so this is the first study. Major complications were seen in Merit, as it was highly used in malignant conditions. Further studies should be performed to compare both stents head to head with a larger sample size.

## Conclusions

The esophageal stent is a minimally invasive procedure that is often used in benign and malignant esophageal disorders. There are numerous treatment options for malignant and benign esophageal conditions, but endoscopic stenting offers significant advantages over other therapies, as they are generally easy and quick to place and safe with a good complication profile. We conducted a retrospective study of 67 patients who underwent Boston Scientific Wallflex and Merit Medical Endotek for benign and malignant esophageal conditions. Our study showed that the Merit stent was mainly used, and the major indication of stent placement was a malignant condition. Major complications were seen when the reason for stent placement was a malignant condition, the diameter was 19 mm, the length was 120 mm, and the use of Merit stent.
